# Bilateral Spontaneous Perirenal Hemorrhage due to Initial Presentation of Polyarteritis Nodosa

**DOI:** 10.1155/2015/428074

**Published:** 2015-09-01

**Authors:** Hyung-Il Choi, Yang-Gyun Kim, Se-Yun Kim, Da Wun Jeong, Ki-Pyo Kim, Kyung-Hwan Jeong, Sang-Ho Lee, Ju-Young Moon

**Affiliations:** Department of Internal Medicine, Division of Nephrology, College of Medicine, Kyung Hee University, Seoul 02447, Republic of Korea

## Abstract

Spontaneous perirenal hemorrhage (SPH) is uncommon but can be a life-threatening condition which is associated with flank or abdominal pain and hypovolemia. The etiologies of SPH include tumor, vascular disease, and infection. Among the vascular diseases, polyarteritis nodosa (PAN) is common cause of the SPH. However, patients with PAN usually complain of nonspecific symptoms and the incidence of PAN is relatively rare. So, diagnosis is difficult even though tissue biopsy and angiography help to confirm the PAN. Particularly bilateral perirenal hemorrhage is very rare complication in patients with PAN. We reported a case of bilateral perirenal hemorrhage in the patients with PAN who have continued to take exogenous sex hormone.

## 1. Introduction

Spontaneous perirenal hemorrhage (SPH) is a rare but potentially life-threatening condition which is characterized by hemorrhage into perirenal space. It is associated with flank pain, palpable abdominal or flank mass, and hypovolemia which is called* Lenk's triad*. However, imaging modalities such as abdominal ultrasonography or computed tomography (CT) are needed to make a diagnosis because these symptoms are nonspecific [1]. Polyarteritis nodosa (PAN) is a systemic necrotizing vasculitis involving small and medium-sized arteries. The patients with PAN usually showed fever, weight loss, abdominal discomfort, and sometimes more localized symptoms suggested of specific organ involvement with nonspecific laboratory findings with elevated C-reactive protein, leukocytosis, and chronic anemia [2]. The kidney is the most commonly involved organ in PAN, accounting for 65–70% [2]. Nonetheless, bilateral SPH is a very rare finding relative to proteinuria, hematuria, and renal insufficiency among the manifestations of PAN. Here, we report a case of bilateral SPH presented as a first manifestation of PAN.

## 2. Case Presentation

**Figure 1:**
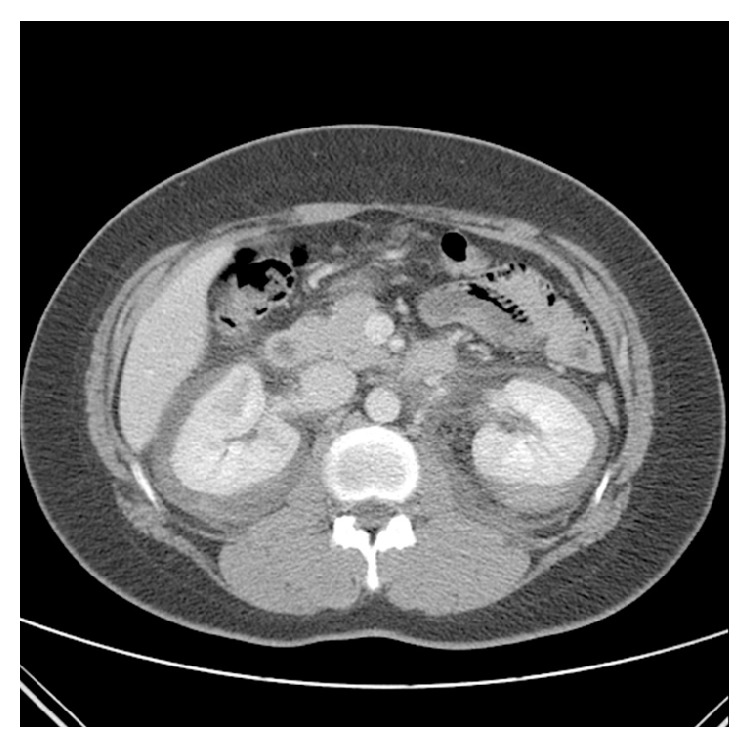
Contrast enhanced CT on the day of admission. CT revealed nonspecific spontaneous bilateral perirenal hemorrhage and delayed enhancing lesion of renal medulla.

**Figure 2:**
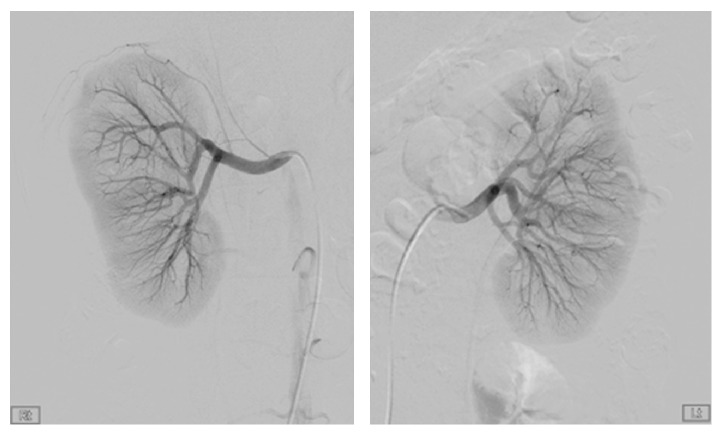
Conventional renal angiography. Renal angiography showed to multiple small aneurysms at the arcuate artery of both renal arteries.

**Figure 3:**
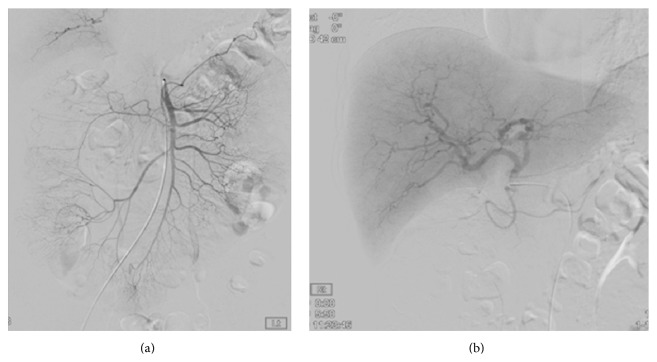
Conventional angiography showed to multiple dot-like small aneurysms, (a) at the jejunal branches of the superior mesenchymal artery and (b) branches of both hepatic arteries.

A 32-year-old woman was admitted to our emergency room with generalized myalgia, mild lower abdominal pain, and flank pain located on both right and left sides. She denied febrile sensation, skin lesion, gross hematuria, and dysuria. She had no history of trauma, severe exercise, and abdomen surgery. She sometimes had been taking oral contraceptive (ethinylestradiol 0.03 mg, drospirenone 3 mg) for last 3 years due to polycystic ovary syndrome (PCOS). Recently, for the purpose of in vitro fertilization, she was taking oral contraceptive for 30 days (estrogen 1.25 mg for 10 days followed by dydrogesterone 10 mg for 10 days and then estrogen 1.25 mg for 10 days). Physical examination revealed the following: blood pressure, 143/91 mmHg; pulse, 95 beats/minute; respiration rate, 20 breaths/minute; and body temperature, 37.3°C. The abdomen was soft, but knocking pain was found over both costovertebral angle. Her blood test showed white blood cell count, 12,900/*μ*L, with absolute neutrophil count, 10300/*μ*L; hemoglobin, 11.1 g/dL; hematocrit, 33.2%; platelet, 330,000/*μ*L; erythrocyte sedimentation rate, 79 mm/hr; C-reactive protein, 7.70 mg/dL; blood urea nitrogen, 13 mg/dL; and creatinine (Cr), 1.1 mg/dL. Urine analysis showed no microscopic hematuria, pyuria, and bacteriuria. Test for hepatitis B surface antigen, human immunodeficiency virus antibody, hepatitis C virus antibody, antinuclear antibody, anti-neutrophil cytoplasmic antibodies, and anti-dsDNA antibody was all negative. To verify the cause of both acute abdominal pain and flank pain, contrast enhanced CT scan was performed. The CT revealed both perirenal hemorrhage and focal ischemic change in upper pole of left kidney and lower pole of right kidney with delayed enhancing of both renal medulla (Figure 1). Renal angiography was performed to determine the cause of hemorrhage. Angiography presented multiple small aneurysms at the arcuate artery of both renal arteries, jejunal branches of the superior mesenteric artery, and branches of both hepatic arteries, which were pathognomonic findings for PAN (Figures 2 and 3). Since there was no evidence of active bleeding, the patient received conservative management with acetaminophen and absolute bed resting without angiographic embolization. Hemoglobin level was maintained from 9.1 g/dL to 10.2 g/dL without transfusion and her pain was relieved spontaneously, and then she was discharged from hospital.

## 3. Discussion

In 1856 Wunderlich firstly described clinical picture of SPH. Acute onset of abdominal or flank pain without a history of trauma is the most common clinical presentation and other features included hypovolemic shock, palpable mass, fever, anemia, and hematuria [3]. Over 10% of patients with SPH develop to hypovolemic shock; therefore early diagnosis and prompt management are essential [4]. Ultrasonography is highly sensitive for detection of perirenal hemorrhage and may be considered as first modality in patients with suspicious SPH. However, ultrasonography alone can lead to misdiagnose hemorrhage as a renal tumor or an abscess and cannot identify the etiology of SPH in most cases [5, 6]. Contrast enhanced CT has a better sensitivity for diagnosis of SPH with a sensitivity of 79~80% and can also find out the etiology of SPH such as renal mass and renal vascular abnormalities [7, 8].

Zhang et al. [9] reported that SPH was associated with tumor (61.5%), vascular disease (17%), and infection (2.4%). Among the vascular diseases, most common cause was PAN (12%). In this meta-analysis, 5 patients (3%) presented with bilateral SPH and all of them were diagnosed with PAN, suggesting that clinicians should keep in mind PAN as a causative disease when they met the patients with bilateral SPH. In Korea, only one case of PAN complicated by renal rupture was reported [10].

According to French Vasculitis Study Group criteria, (1) evidence of arteriographic anomaly, (2) absence of cytoplasmic antibody, and (3) absence of Ear, nose, and throat (ENT) symptom, the study cohort achieved a diagnostic accuracy of 79% for PAN [11]. Although studies about the accuracy of angiography are limited, Stanson et al. revealed that 40–90% of patients with PAN showed typical angiographic findings, including aneurysms, occlusive disease, or ectasia [12].

In our case, the patient was diagnosed of PCOS before 15 years ago and had taken an oral contraceptive for 3 years and recently received the hormone therapy (estrogen and dydrogesterone) for the purpose of ovulation and pregnancy. Although direct role of oral contraceptive and hormone therapy on the arterial thrombosis has not been clear yet, several studies showed that exogenous sex hormone has a prothrombotic effect [13, 14]. Also estrogens in women with PCOS promoted immune dysregulation by stimulating the secretion of IL-4 in Th2 lymphocytes, IL-1 in monocytes, IL-6 in T lymphocytes, and interferon-*γ* in Th1 lymphocytes [15]. Also, the patients with PCOS have elevated serum VEGF, which is an endothelial cell mitogen promoting angiogenesis and increasing vascular permeability [16]. Therefore, it could be possible that estrogen excess status with exogenous sex hormone stimulated the immune system and upregulated VEGF and then caused bleeding from PAN lesion. After hospital admission, the exogenous hormone stopped because of the reasons mentioned above. She did not exhibit typical PAN symptoms, such as arthralgia and fatigue, or typical signs, such as skin lesions, renal insufficiency, and hypertension. In addition, although fever occurred before the admission, there was no fever during hospital days. Therefore, we observed the disease course without systemic medication, a corticosteroid which is an initial treatment of typical PAN [17]. Three months later after stopping the exogenous hormone, her symptoms were completely improved and ultrasonography finding revealed decrease size of hematoma in PAN lesion. Although more observations were required, the patient did not visit the outpatient clinic.

In summary, SPH is clinically uncommon but is critical disease because it can be a life-threatening condition, so clinicians need to diagnose the disease exactly and know the causes of SPH for a forcible treatment. We report rare case of bilateral SPH in PAN lesions, which is suspected to be caused by exogenous sex hormone.
